# Effect of the Rovatti Method^®^ (Physiotherapeutic Scoliosis-Specific Exercises) in an Adolescent Patient with Idiopathic Scoliosis: A Case Report

**DOI:** 10.3390/reports8030171

**Published:** 2025-09-06

**Authors:** Marco Rovatti, Emanuele Rovatti, Guido Belli, Niccolò Baldoni, Pasqualino Maietta Latessa

**Affiliations:** 1 Rovatti Plan Medical Center, Cassano D’Adda, 20062 Milan, Italy; marco@rovattiplan.it (M.R.); info@rovattiplan.it (E.R.); 2 Department of Life Quality Studies, University of Bologna, 47921 Rimini, Italy; niccolo.baldoni2@unibo.it (N.B.); pasqualino.maietta@unibo.it (P.M.L.)

**Keywords:** adolescent idiopathic scoliosis (AIS), physiotherapeutic scoliosis-specific exercises (PSSE), case report, Rovatti Method^®^

## Abstract

**Background and Clinical Significance**: The study aims to investigate the application of the Rovatti Method^®^ in improving Cobb angles, angle of trunk rotation (ATR), aesthetics, and quality of life in the conservative treatment of adolescent idiopathic scoliosis (AIS); **Case Presentation**: The case concerns the application of the Rovatti Method^®^ in treating a 13-year-old girl with mild right thoracolumbar AIS. This method involves the use of elastic bands designed to guide and enhance proprioceptive and mechanical stimuli during the patient’s active self-correction exercises. The treatment lasted 7 months; a radiographic control showed an improvement in the right thoracolumbar curve, with Cobb angles decreasing from 21° to 14°, ATR from 10° to 8°, TRACE (Trunk Aesthetic Clinical Evaluation) decreasing from 8 to 4 points, and the Scoliosis Research questionnaire (SRS-22) improving from 2.27 to 3.05 points. **Conclusions**: Concerning this hypothesis-generating observation case, the Rovatti Method^®^ may represent a kinesiological approach for the treatment of AIS, potentially contributing to improvements in Cobb angles, ATR, aesthetics, and quality of life.

## 1. Introduction and Clinical Significance

Adolescent idiopathic scoliosis (AIS) is a three-dimensional structural deformation of the spine, typically affecting patients aged 10 years to skeletal maturity [1,2]. AIS tends to develop prominently and rapidly at the onset of puberty and represents the most frequent type of scoliosis, with 80% of total cases [1,3,4]. During puberty, growth initially occurs in the limbs, leading to temporary body disproportion, where the torso appears proportionally shorter. Subsequently, growth progresses in the axial skeleton, a phase during which scoliosis is more likely to develop or worsen [4,5].

This condition affects up to 2–3% of young people around the world and progresses more frequently in females, with the female-to-male ratio increasing as Cobb angles rise: 1.3:1 for 10–20°, 5.4:1 for 20–30°, and 7:1 for angles above 30° [5]. Additionally, AIS shows a higher prevalence among females in Nordic countries, suggesting possible environmental factors in AIS pathogenesis [6,7].

Although the etiology of AIS remains undefined, certain pathogenic and biomechanical characteristics have been identified [7]. Spinal growth is a very dynamic and nonlinear process during which several factors can negatively influence the hierarchy and harmony of the progression [8]. In particular, visual problems [9], vestibular alterations [10], stomatognathic diseases [11], and metabolic [12] and genetic factors [13] have been identified as facilitating the asymmetrical development of the spine. Consequently, there is an imbalance between the growth of anterior and posterior vertebral elements. Vertebral bodies grow faster than posterior elements, deforming to expand and causing pathological rotation, leading to asymmetrical modification of the rib cage [14,15]. In addition, people with AIS have an erroneous perception of the gravitational vertical, resulting in possible dysfunction of trunk graviceptors and subjective postural alignment [16]. The consequent upright posture alters the spinal loading, which is physiologically different in humans compared to other primates [14]. Human vertebrae are anatomically ill-equipped to withstand dorsally oriented shear forces, which can exacerbate preexisting vertebral rotation, promoting scoliosis progression and reducing rotational control by interapophyseal joints. Thus, scoliosis may result from a complex and progressive process that negatively influences the morphological structure and stability of the spine [8,14,15]. The treatment of AIS can be surgical or conservative, in relation to the magnitude of pathology and subject’s profile [1,17]. A conservative approach includes observation, physiotherapeutic scoliosis-specific exercises (PSSE), and braces [18]. The effectiveness of braces has been widely reported, and the utilization is mainly recommended for AIS ranging between 25° and 40° (moderate scoliosis) [19]. Differently, PSSE with a focus on active self-correction, spinal stabilization, and cognitive behavioral education is suggested in AIS lower than 25° (mild scoliosis), but the efficacy remains unclear [20].

Conservative AIS treatment using PSSE is described in the literature, and several methods are presented, even if the absence of high-quality studies is underlined [21,22,23]. Contrary to popular belief, no evidence suggests surgical treatment is superior to conservative AIS management. Indeed, no significant differences were observed in Scoliosis Research Society questionnaire (SRS-22) scores over an 8-year follow-up between AIS patients with curves ≥40° treated surgically or non-surgically [24]. Surgery, associated with long-term complications, should be approached cautiously [25,26]. Consequently, conservative AIS treatment should also be considered for patients with surgical indications.

According to SOSORT (International Society on Scoliosis Orthopaedic and Rehabilitation Treatment), AIS conservative treatment should focus on active self-correction of the spine in all three dimensions through brace and/or PSSE in relation to the severity of scoliosis [1,27]. This principle underpins the Rovatti Method^®^, which employs elastic bands for three-dimensional spinal correction [27,28]. The method is an Italian conservative approach for AIS that was introduced around 1990 by Emanuele Rovatti and developed in the following years [28]. Since the method aligns with the literature guidelines, this study aims to analyze the application of Rovatti Methods^®^ in AIS conservative treatment of a case study and describe this approach.

The selection of this specific case was related to multiple reasons: the magnitude of AIS (representing a mild scoliosis with medical prescription of PSSE), the positive and continuous participation in the training program for more than 6 months, the self-awareness of body image, and the strong willpower in improving the initial condition [27,29].

## 2. Case Presentation

This study involved a female patient with a diagnosis of AIS and was conducted according to the CaRe checklist (Case Reports guidelines). The patient presented X-rays and a medical report documenting right thoracolumbar scoliosis (T10-L3) of 21° Cobb, with Risser 2/3+. Clinical examination data included age 13.4 years, height 156 cm, sitting height 81 cm, weight 42 kg, and menarche at age 11.7. Since the participant was younger than 18 years old, a parent was informed and gave voluntary consent to take part in the study according to the ethical principles of the Declaration of Helsinki.

The functional assessment included angle of trunk rotation (ATR), quality of life, and aesthetics [29,30]. ATR was measured using a Bunnel scoliometer during the forward bending test, resulting in 10° [31]. Quality of life was assessed using the Scoliosis Research Questionnaire (SRS-22, Italian version), yielding a mean score of 2.27 [32]. Aesthetic evaluation using the TRACE (Trunk Aesthetic Clinical Evaluation) scale gave a score of 8 [33]. ATR, SRS-22, and TRACE represent useful tools for screening people with mild AIS and are commonly used to obtain information about subjective and objective body shape [29,34]. The minimal clinically important difference (MCID) for Cobb angle and mean SRS-22 domain scores are 5° and 0.40–0.70, while the minimal detectable change (MDC) for TRACE and ATR are 3–4 and 3°, respectively [33,34,35,36,37]. The clinical and functional evaluations were led by a physician (with specialization in orthopedics and physical medicine) along with a physical therapist pre- and post-intervention

The rehabilitation program, based on the Rovatti Method^®^ principles, lasted 7 months, culminating in a follow-up radiographic evaluation to reassess Cobb angles.

### 2.1. Methods

**Figure 1:**
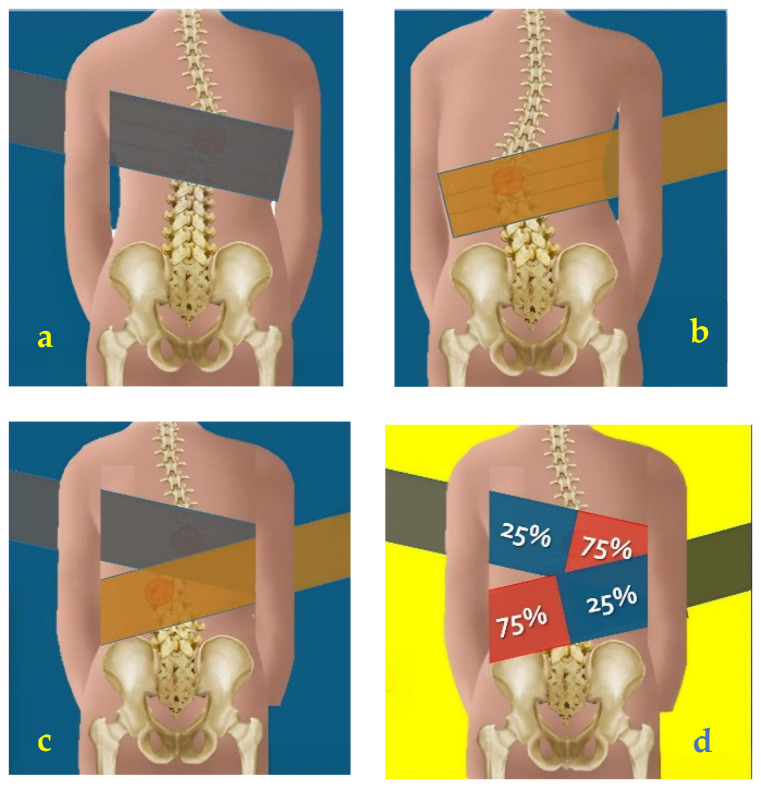
The application of elastic band: (**a**) dorsal curve positioning; (**b**) lumbar curve positioning; (**c**) double curve positioning; (**d**) 75% above the hump, 25% anchorage.

The Rovatti Method^®^ distinguishes itself in conservative AIS treatment by using elastic bands to shape the spine, guiding patients toward active self-correction and internalizing a physiological and correct body schema [28]. This method involves active self-correction exercises enhanced by proprioceptive and mechanical stimuli from force vectors generated by elastic bands wrapped around the patient to provide continuous three-dimensional spinal stimulation [21,27]. The elastic band is tensioned similarly to a functional bandage, with approximately 75% tension applied above the hump to provide a proprioceptive stimulus in the direction of correction and 25% on the opposite side, acting as an anchor (Figure 1). To determine the percentage of tensioning, 100% tension is applied by stretching the band fully with both hands and then reducing it by 1/4 to obtain approximately 75%, or by 3/4 to obtain 25%. Several studies in literature, particularly those focused on TheraBands^®^, have investigated the relationship between the degree of elongation and the corresponding resistance generated, providing reference values and methods for standardizing the applied tension [38]. The current training protocol was performed using TheraBand^®^ latex elastic band (Hygienic Corp, Akron, OH, USA), starting from the silver band and progressing to the gold band [39].

Correction begins by restoring the physiological curves above the sagittal plane, then in the coronal plane with a lateral tilt, and finally with a rotation in the transverse plane.

Exercises include two components: elastic band positioning and tensioning, and patient-performed self-correction [22,23]. Studies have shown that active correction can reduce spinal deformities and improve SRS-22 scores [1,27].

**Figure 2:**
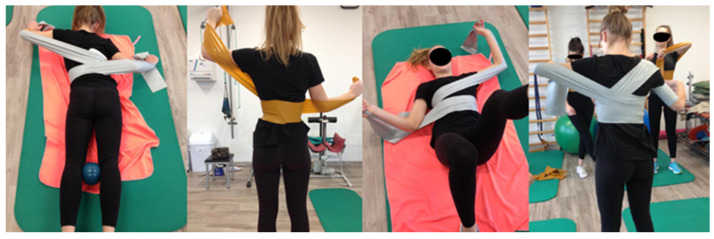
Different exercises with elastic bands according to the Rovatti^®^ Method.

Initially, as per the Rovatti Method^®^ protocol, the patient performed exercises without elastic bands to enhance body awareness and self-correction capabilities. Elastic bands were then introduced for active correction exercises (Figure 2).

All exercises followed the principles of the Rovatti Method^®^ and were tailored to the individual patient’s postural and motor characteristics. Exercises were classified as direct (with elastic band force vectors aligned with the direction of correction) or indirect (with vectors opposing the corrective direction). In direct exercises, the patient performed a brief, sharp inhalation followed by a long, slow exhalation, maintaining the corrective position for at least 20 s to promote cortical integration. Correction was initiated in the sagittal plane: thoracic curves were typically addressed with flexion movements to restore physiological kyphosis, whereas lumbar corrections employed extension to recover natural lordosis. The goal in both cases was to approach physiological sagittal alignment as much as possible [30].

In indirect exercises, particularly for thoracic scoliosis, correction occurred during the inspiratory phase. Patients were instructed to focus airflow into the concave hemithorax, facilitated through tactile stimulation provided by the therapist.

All exercises concluded with a controlled return phase, reversing the corrective sequence—typically derotation, lateral tilt, and then sagittal extension—to enhance proprioceptive awareness and prevent unintentional return to dysfunctional patterns.

Lumbopelvic stabilization was emphasized, especially in thoracic curves, and was facilitated through pre-activation strategies such as adductor engagement using a ball between the knees to promote core activation. Core strengthening has been shown to reduce lumbar curve Cobb angles in AIS patients [40].

Additional tools such as sticks, mirrors, and proprioceptive aids were used as needed. Exercise selection was individualized according to specific protocol guidelines and patient needs.

Each treatment session lasted one hour and was scheduled twice a week (occasionally three times) during the afternoon (around 4.00–5.00 PM) in the same gym with comfortable environmental conditions, allowing the adolescent to balance therapy with daily commitments [41]. Sessions were held in homogeneous groups, where AIS patients followed personalized rehabilitation plans under physiotherapist supervision [21,22]. Each session included approximately 12 exercises lasting 5 min each, encompassing both execution and rest phases. Within each 5-min interval, the active part of the exercise lasted about 30 s per repetition, with rest periods generally set at half the active duration. For more intense exercises, the rest period could be extended to match the active phase (1:1 ratio). The sessions were led by two specialized physical therapists with more than 15 years of experience in scoliosis treatment (a summary of the protocol is shown in Table 1, while the complete training protocol and exercise are provided in Supplementary File S1).

This approach facilitated discussions on biopsychosocial challenges related to the AIS condition, promoting therapy adherence and a shared growth journey [42]. Group therapy reduced economic burdens for families due to long-term treatment needs.

### 2.2. Results

**Figure 3:**
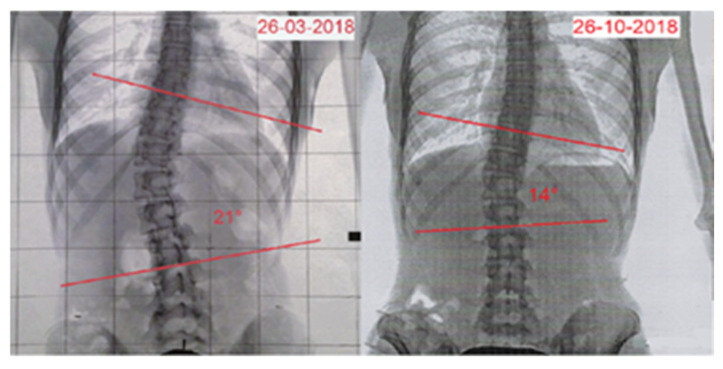
Rx before and after treatment.

**Figure 4:**
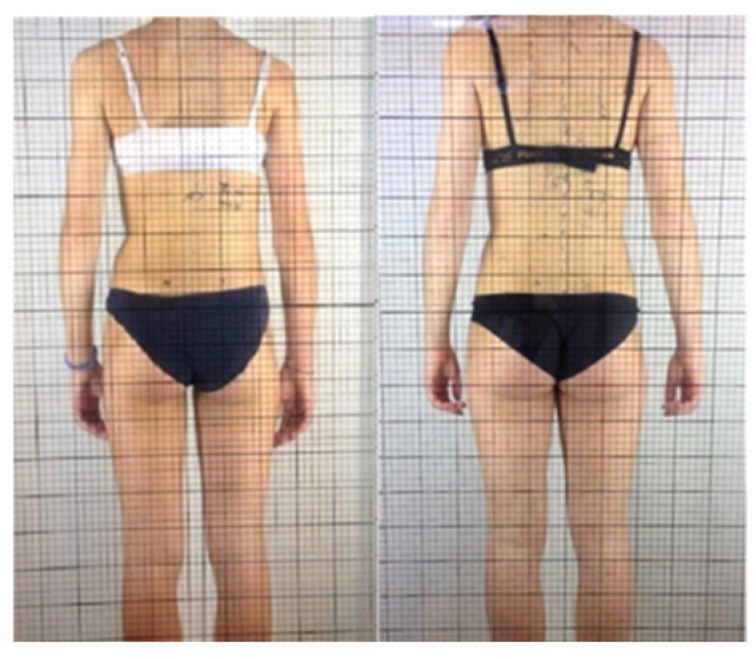
Before and after treatment images used for TRACE scale.

After 7 months, the patient underwent a follow-up X-ray (Figure 3), which showed an improvement in the right thoracolumbar curve Cobb angle, decreasing from 21° to 14° (Risser 3/4+), corresponding to a 33.3% reduction. Clinical examination data were updated as follows: age 13.11 years, standing height 157 cm, sitting height 82 cm, and weight 45 kg. ATR, measured through the bending test, showed an improvement from 10° to 8°, indicating a 20% decrease. The SRS-22 questionnaire score increased from 2.27 to 3.05 points, indicating a positive change (25.5% improvement). Aesthetic evaluation using the TRACE scale improved significantly, from 8 to 4 points, showing a 50% decline in perceived asymmetry (Figure 4) (Table 2).

## 3. Discussion

The Rovatti Method^®^ stands out for its use of elastic bands. It is hypothesized that the mechanical and proprioceptive stimuli provided by these bands positively influence improvements in Cobb angles, ATR, and aesthetics (TRACE).

The elastic bands used in the Rovatti Method^®^ act not only as mechanical support but generate proprioceptive force vectors that actively stimulate the spine toward correction. These vectors can be of two types:

1. Direct vectors: the tension of the elastic band is applied in such a way as to guide the body in the direction of correction, providing support that facilitates reaching the correct position. This approach is useful in patients who need more structured guidance and constant reinforcement of self-correction [43,44].
2. Indirect Vectors (Reactive Neuromuscular Training—RNT): in this case, the resistance of the elastic band is applied in the opposite direction to the correction, inducing an active compensatory response from the patient [45]. This type of stimulus exploits the principle of neuromuscular hyper-correction, forcing the patient to counteract the force of the rubber band and respond with a more intense active correction. The RNT method is particularly effective in those patients who respond better to counterfactual stimuli, improving neuromuscular control and active stabilization of the spine [46,47,48].

In the present work, all postural evaluations improved after the training period. Cobb angle, ATR, SRS-22, and TRACE scores enhanced, ranging from 20% to 50%, and the results were close to or beyond the minimal clinically important differences (MCID) for each test [34]. Even if a measurement error cannot be excluded and the accuracy of the tests (in particular ATR and TRACE) is related to professional experience, it is reasonable to suppose that the combination of a physician and a physical therapist with strong expertise about scoliosis could limit these errors during assessment phases. Furthermore, the concurrent modification in Cobb angle (measured with X-ray, the gold standard for scoliosis assessment) and other clinical and functional tests are directed toward positive improvements [27,42]. In conclusion, the training with this approach may have contributed to the reported results.

### 3.1. Advantages of the Rovatti Method

A specific advantage of the Rovatti Method^®^ is the transformation of a purely manual approach, in which the therapist applies manual stimuli to the patient, to an active method based on exercise using elastic bands.

This change brings several benefits [42,49,50]:

- Better cost–benefit ratio, since it is not a one-to-one, therapist-patient, exclusively manual treatment, allowing work in small groups. This approach reduces costs and permits treatment to be extended in the medium to long term, a key aspect for a condition such as scoliosis, which requires a continuous approach over time.
- Ability to create small groups, fostering mutual support among patients and improving the therapeutic experience, both in terms of motivation and effectiveness.
- Increased therapeutic adherence, thanks to a method that actively involves the patient in the rehabilitation process, making them more aware and responsible for their own postural correction. The corrective approach in the Rovatti Method^®^ is based on three-dimensional management of scoliosis, intervening in the different planes of movement:
  (a) Sagittal plane: The main objective is to restore the physiological curves of the spine, providing balance between lumbar lordosis, dorsal kyphosis, and cervical lordosis [1,51]. Scoliosis, being a three-dimensional deformity, often alters the natural alignment of the vertebral curves, making their restoration essential to improve the biomechanics and stability of the spine [27].
  (b) Frontal plane: Correction aims to reduce Cobb’s angle, improving symmetry of the spine and rib cage [21,22].
  (c) Transverse plane: Proprioceptive stimulation via elastic bands facilitates vertebral de-rotation, counteracting the torsional asymmetries typical of idiopathic scoliosis [44,46].

Even if the debate about the role of exercise in the management of AIS is still open, the international guidelines about AIS treatment promote this three-dimensional approach and the role of self-correction [20,21,22]. Recently, the review of Romano et al. evidences the heterogeneity of the studies and the need for further randomized controlled trials (RCTs) in order to better understand the role of PSSE in improving aesthetics and quality of life [23]. Actually, PSSE seems to have different benefits in relation to specific methodology with SEAS and Schroth approaches (both focusing on previous points), evidencing the best results [18,21].

Consequently, the results achieved may be attributed to the common principles of the Rovatti Method^®^ with these approaches. In addition, more factors could be considered. Certainly, the constant presence of a physiotherapist supervising each session ensured proper, effective, and conscious exercise execution [50].

The integration of proprioceptive input, force vectors, and neuromuscular activation strategies allows for an adaptive and individualized approach, optimizing the therapeutic response according to the characteristics of the patient [46,47].

Additionally, the group setting provided a supportive environment for AIS patients to share experiences and challenges. This social interaction likely enhanced therapy adherence and motivation [42].

### 3.2. Limitations of the Rovatti Method

Like any corrective approach, the Rovatti Method^®^ requires good motor control, an essential element for the patient to actively apply the required postural corrections.

For this reason, it is essential to assess the patient’s subjective ability, considering the level of body perception and awareness. In the early stages, individual work on body awareness and education about self-correction may be necessary before placing him or her in a group work setting. In the current study, the first weeks were performed individually in order to learn the previous corrective elements.

This aspect makes the initial phase of treatment particularly important to ensure maximum benefit in the long term and to avoid incorrect compensations that could reduce the effectiveness of the intervention.

### 3.3. Limitations of the Study

Since the current work describes a single case, the results must be interpreted with the limits related to this specific setting. The assessment tools, measurement errors, and subjective evaluation during this process could have influenced the results. In addition, the role of growth-related changes, the curve progression process, and patient motivation must be considered. Moreover, although the study presents results after 7 months of intervention, the absence of long-term follow-up data limits the interpretation of clinical relevance. Anyway, this experience could be a positive contribution to the future investigation of the method. Further large-scale studies are needed to confirm these findings. In particular, randomized controlled trials (RCTs) that compare this method with SEAS and Schroth approaches should represent the subsequent step to better investigate this contribution in conservative management of AIS, ideally including a 12–24 month follow-up to assess maintenance of results.

### 3.4. Patient Perspective

The participation in the Rovatti Method^®^ training program was positively experienced by the patient. The continuous presence during the 7-month period allowed them to improve their self-body awareness, create a strong and confident relationship with their physical therapist, and enhance the human connections in personal contexts (school, family…). The patient learned to serenely accept his condition and behaved in order to increase the body and mind wellness. The satisfaction with treatment was underlined by the SRS-22 score.

## 4. Conclusions

The use of elastic bands in the Rovatti Method^®^ might represent a possible innovation in the conservative treatment of scoliosis, as it is not limited to biomechanical support but actively stimulates neuromotor control. The combined use of direct and indirect vectors allows the corrective response to be modulated according to individual needs, while three-dimensional management of scoliosis ensures functional restoration of physiological curves. These factors might have contributed to enhancing aesthetics and quality of life.

Due to its potential to facilitate teamwork and reduce therapeutic costs over time, this method may represent an affordable, engaging, and potentially effective approach, contributing to improved treatment quality and patient adherence. However, its effectiveness could depend on appropriate patient selection and adequate motor control preparation, which appear to be important factors in optimizing outcomes. These positive results should be considered as a hypothesis-generating observation setting and might lead to future investigation about the efficacy of the Rovatti Method^®^.

## Figures and Tables

**Table 1:** Summary of the protocol administered.

**Session duration**	60 min
**Frequency**	2×/week (occasionally 3×)
**Structure**	~12 exercises, 5 min each – Active phase: ~30 s/rep – Rest 1:2 ratio (half the active duration), or 1:1 for more intense exercises
**Principles**	Rovatti Method^®^: self-correction + elastic bands (direct/indirect), guided breathing, controlled return
**Focus**	Sagittal alignment, lumbopelvic stabilization, core strengthening
**Setting**	Small AIS groups, supervised by experienced physiotherapists, comfortable environment

**Table 2:** Results pre- and post-treatment after 7 months.

Time	Age	Standing Height	Sitting Height	Weight	Cobb Angle	Risser	ATR	SRS-22	TRACE
Pre	13.4	156 cm	81 cm	42 kg	21°	2/3+	10°	2.27	8
Post	13.11	157 cm	82 cm	45 kg	14°	3/4+	8°	3.05	4

## Data Availability

The data presented in this study are available on request from the corresponding author.

## Supplementary Materials

The following supporting information can be downloaded at: https://www.mdpi.com/article/10.3390/reports8030171/s1, Table S1: Neuromotor Progression – Rovatti Method (Dorsal Curve).
