# Supplementary material for: Effect of the Rovatti Method® (Physiotherapeutic Scoliosis-Specific Exercises) in an Adolescent Patient with Idiopathic Scoliosis: A Case Report
Source: Reports (MDPI). 2025 Sep 6;8(3):171. doi: 10.3390/reports8030171 (PMC12452423; doi:10.3390/reports8030171)
Supplement: Supplementary file 1 [file reports-08-00171-s001.zip › reports-3786010-supplementary.pdf]

Case Report

Effect of the Rovatti Method® (Physiotherapeutic Scoliosis - Specific Exercises) in an Adolescent Patient with Idiopathic Scoliosis: A Case Report

Marco Rovatti [1], Emanuele Rovatti [1], Guido Belli [2,*], Niccolò Baldoni [2] and Pasqualino Maietta Latessa [2]

[1] Rovatti Plan Medical Center, Cassano D'Adda, Milan 20062, Italy; marco@rovattiplan.it (M.R.); info@rovattiplan.it (E.R.)
[2] Department of Life Quality Studies, University of Bologna, 47921 Rimini, Italy; niccolo.baldoni2@unibo.it (N.B.); pasqualino.maietta@unibo.it (P.M.L.)
* Correspondence: guido.belli@unibo.it

Table S1 Neuromotor Progression – Rovatti Method (Dorsal Curve).

Neuromotor Stage	Exercise	Description	Figure / Photo
**Supine / Breathing Control**	1- Directed Costal Breathing	Stimulation of inspiration by focusing attention on the therapist's hand, resulting in costal correction (directed breathing). Different hand placements.	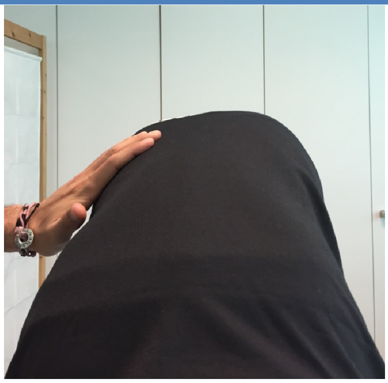
**Prone**	2- Prone band	Prone: recreate sagittal physiology (thoracic kyphosis + lumbar lordosis), then lateral deviation and derotation with hand push. Direct or indirect stimulus.	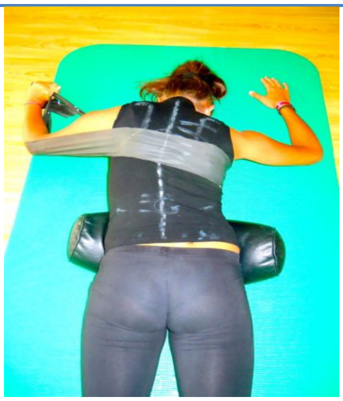
**Side-lying / Side bridge**	3- Side bridge band	Side bridge position, core activation. The band wraps around the dorsal hump creating a 3D corrective stimulus.	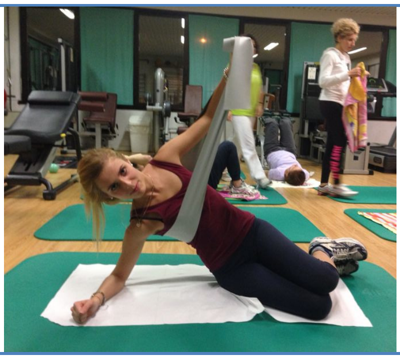

**Quadrupedic /4 points kneeling**	4-	Quadruped position	Six support points (hands, knees, feet). The band is held to the floor by the hand. The stimulus can be corrective or dysfunctional.	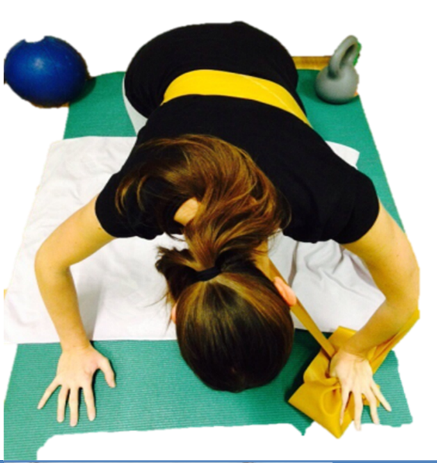
**Sitting (fitball)**	5-	Fitball sitting band	Sagittal recovery, then lateral deviation and derotation. Fitball opens the ilio-lumbar angle and limits compensations. Direct or indirect stimulus.	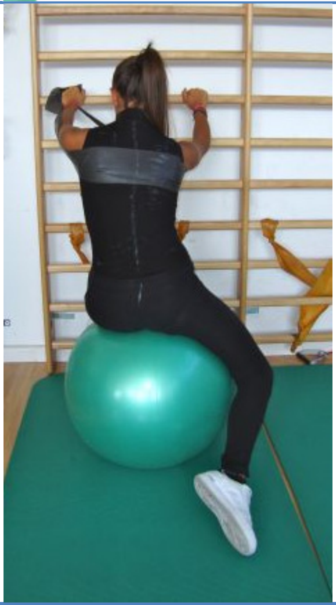
**Half kneeling**	6-	Half Kneeling band	Intermediate position with 3 supports (foot-knee-foot). Stabilizes the pelvis. The band stimulates lateral deviation and derotation toward correction.	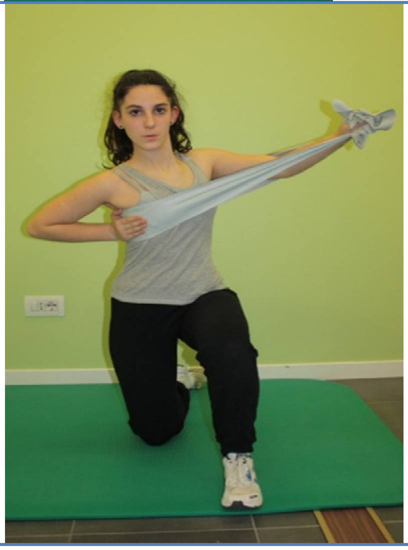

**Standing**	7-	Wall-Fix Band Correction	Hands fixed to wall. Band stimulates lateral deviation and derotation. Elbow facilitates and varies vectors.	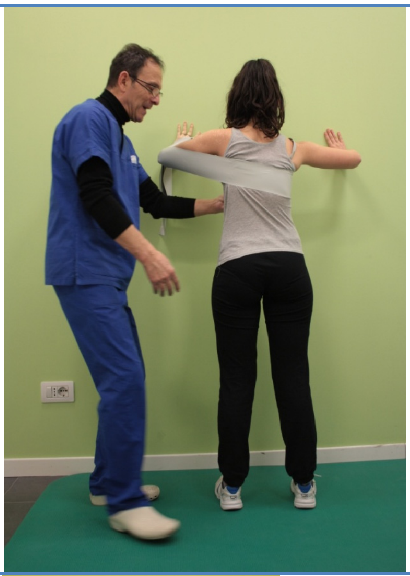
**Standing**	8-	Semi-Bending Band Correction	Semi-bending: the band wraps the dorsal hump with a direct corrective stimulus. Greater activation of paravertebral muscles.	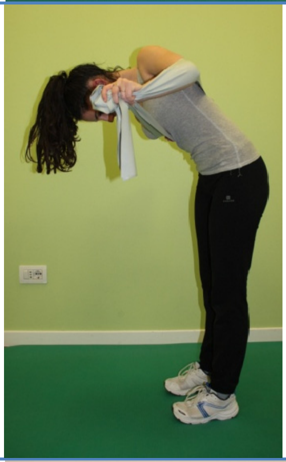
**Standing**	9-	Band Correction with Stick	Correction with stick as external fixation and core activation via ball. Direct or indirect stimulus.	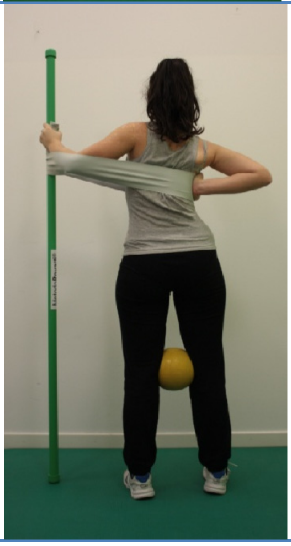

**Standing**	10- Indirect Correction + Breathing	Indirect correction: opposite vectors + directed breathing. Patient corrects thoracic kyphosis/lumbar lordosis, then lateral deviation + derotation.	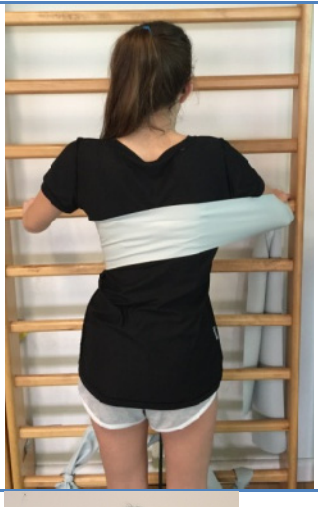
**Standing**	11- Arm Band with Core Activation	Band from arm → scapular stabilizer activation, passes over rib cage and hump. Ball between legs activates core. Direct vectors for lateral deviation, indirect for derotation.	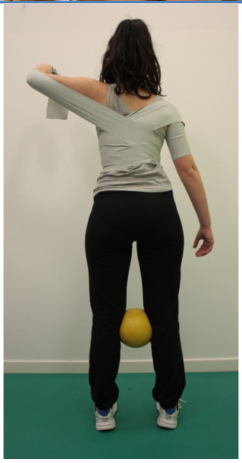
**Standing**	12- Pectoralis Push with Stick	Stick as fixed point, downward push activates pectoralis and facilitates derotation.	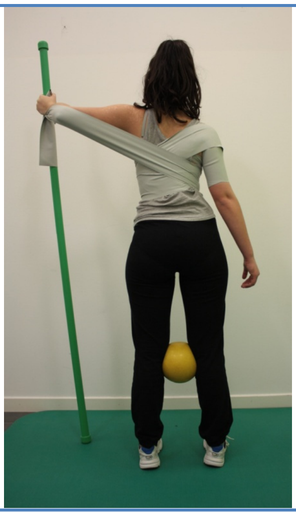
